# The National Cancer Institute’s Cancer Disparities Research Partnership Program: a unique funding model 20 years later

**DOI:** 10.1093/jnci/djad173

**Published:** 2023-09-14

**Authors:** C Norman Coleman, Rosemary Wong, Daniel G Petereit, Patrick D Maguire, Dwight E Heron, Michael Steinberg, Yadvindera Bains, Bhadrasain Vikram, Patricia Angelis, Alicia A Livinski, Mack Roach, Frank S Govern

**Affiliations:** Radiation Research Program, Division of Cancer Treatment and Diagnosis, National Cancer Institute, National Institutes of Health, Rockville, MD, USA; Radiation Research Program, Division of Cancer Treatment and Diagnosis, National Cancer Institute, National Institutes of Health, Rockville, MD, USA; Monument Health Cancer Care Institute, Rapid City, SD, USA; Coastal Carolina Radiation Oncology (Novant-New Hanover Regional Medical Center Radiation Oncology), Wilmington, NC, USA; Bon Secours Mercy Health System, Youngstown, OH, USA; Department of Radiation Oncology, David Geffen School of Medicine at the University of California Los Angeles, Los Angeles, CA, USA; Baylor Scott & White Clinic, College Station, TX, USA; Radiation Research Program, Division of Cancer Treatment and Diagnosis, National Cancer Institute, National Institutes of Health, Rockville, MD, USA; Radiation Research Program, Division of Cancer Treatment and Diagnosis, National Cancer Institute, National Institutes of Health, Rockville, MD, USA; National Institutes of Health Library, Office of Research Services, Office of the Director, National Institutes of Health, Bethesda, MD, USA; Radiation Research Program, Division of Cancer Treatment and Diagnosis, National Cancer Institute, National Institutes of Health, Rockville, MD, USA; Departments of Radiation Oncology and Urology, University of California San Francisco, San Francisco, CA, USA; Radiation Research Program, Division of Cancer Treatment and Diagnosis, National Cancer Institute, National Institutes of Health, Rockville, MD, USA

## Abstract

The burden of cancer and access to effective treatment are not experienced equally by all in the United States. For underserved populations that often access the health-care system when their cancers are in advanced disease stages, radiation oncology services are essential. In 2001, the National Cancer Institute’s (NCI’s) Radiation Research Program created and implemented the Cancer Disparities Research Partnership Program (CDRP). CDRP was a pioneering funding model whose goal was to increase participation of medically underserved populations in NCI clinical trials. CDRP’s Cooperative Agreement funding supported for awardees the planning, development, and conduct of radiation oncology clinical research in institutions not traditionally involved in NCI-sponsored research and cared for a disproportionate number of medically underserved, health-disparities populations. The awardee secured and provided support for mentorship from 1 of 2 NCI comprehensive cancer centers named in its application. Six CDRP awards were made over two 5-year funding periods ending in 2013, with the end-of-program accomplishments previously reported. With the current focus on addressing equity, diversity, and inclusion, the 6 principal investigators were surveyed, 5 of whom responded about the impact of CDRP on their institutions, communities, and personal career paths. The survey that was emailed included 10 questions on a 5-point Likert scale. It was not possible to collect patient data this long after completion of the program. This article provides a 20-year retrospective of the experiences and observations from those principal investigators that can inform those now planning, building, and implementing equity, diversity, and inclusion programs.

Addressing inequality in access to and use of cancer care is receiving long-overdue and well-deserved attention as part of the National Institutes of Health’s (NIH’s) Equity, Diversity and Inclusion Program (1). More than 2 decades ago, the National Cancer Institute’s (NCI’s) Radiation Research Program within the Division of Cancer Treatment & Diagnosis recognized disparities in cancer care as an important problem to address. This recognition resulted in efforts to increase access to and participation in radiation oncology and multimodality clinical trials for medically underserved populations in the United States. Radiation oncology was emphasized because the health-disparities populations generally experience advanced cancers that often require radiation as a component of care. Furthermore, the need to access experts offered an opportunity to evaluate the use of the NIH’s novel TELESYNERGY telemedicine system.

In 2002, the Division of Cancer Treatment & Diagnosis’ Radiation Research Program developed the Cancer Disparities Research Partnership Program (CDRP), which was a unique, pioneering program to pilot a new strategy whose goal was to increase access to and accrual in NCI clinical trials by underserved and health-disparities communities. CDRP implemented a novel funding approach by which the primary cooperative agreement (U56) was awarded directly to community-based hospitals experiencing cancer care disparities. The funding was provided to establish the infrastructure the community-based institution required for its patients to access NCI-sponsored radiation oncology and other cancer clinical trials. The primary awardee was required to form a partnership with an academic cancer center, which served as a mentor organization. Two potential awardees were required in the grant application because NCI was uncertain any would accept. A portion of the awardee’s funding, $100 000 per year, was provided to the primary mentoring institution for the time spent mentoring (2). This model was unique in that the funding flowed in the opposite direction to historical funding models, where academic cancer centers would receive all funding, and then distribute funds to its local health-disparities communities (Figure 1, A). By program design, the CDRP empowered and funded programs—without a history of participating in NCI or NIH grants—to see whether and how those institutions embedded within and providing care to the underserved community could build research programs that enabled access to clinical cancer research along with a committed mentor (Figure 1, B).

**Figure 1.**
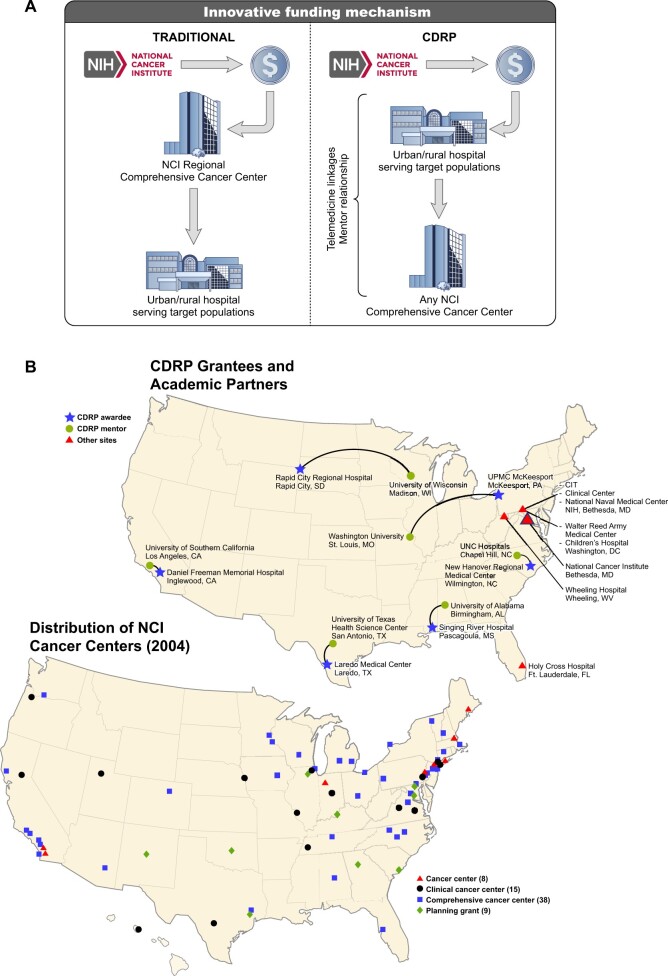
The CDRP offered a unique model that turned the funding paradigm upside down. **A)** The funding paradigm provided the grant award to the CDRP community, with funds then flowing to the NCI cancer center. **B)** Geographic gap that the CDRP filled, including the distribution of the NCI cancer centers in 2004 and the CDRP recipients, including grantees, partners, and a TELESYNERGY pilot program. This pilot involved the NCI Radiation Oncology Branch plus the National Naval Medical Center in Bethesda, Maryland, partnering with Holy Cross Hospital in Fort Lauderdale, Florida, and Wheeling Hospital in Wheeling, West Virginia. CDRP = Cancer Disparities Research Partnership Program; CIT = Center for Information Technology; NCI = National Cancer Institute; UNC = University of North Carolina.

Associated goals of the program included 1) helping the new grantee build the necessary infrastructure and environment at the institutions so that they can become long-term, credible partners for NCI-sponsored radiation oncology and other clinical trials research; 2) increasing the number of clinical and translational scientists working at institutions that serve populations experiencing cancer-related health disparities; 3) fostering and supporting the development of long-term research mentoring partnerships with established NCI-affiliated research centers; and 4) helping bring the grantee institutions up to a level where they could submit competitive applications for future research grants in clinical trials and biomedical research.

A pioneering feature and goal of the CDRP was the use of telemedicine. In 2002, when the CDRP began, telemedicine was just emerging. The CDRP piloted a telemedicine system developed jointly by NCI and the NIH Center for Information Technology (notably, Kenneth Kempner) called TELESYNERGY, which had strong imaging capability. The use of telemedicine assisted in building and solidifying the grantee-mentor relationship—often despite great distances—to help expand clinical and translational research and build relationships between investigators and institutions. For the CDRP, TELESYNERGY was used to train staff, facilitate tumor board conferences, plan treatment, conduct follow-up consultation and second opinions, and generally share ideas between the grantee and mentor institutions.

In addition, the patient navigator program Harold Freeman, MD, created was added to the CDRP U56 cooperative agreement (3). The goals were to contribute to increased accrual into clinical trials among the underserved communities the primary awardee served by providing culturally appropriate education, assistance, and “navigation” through the complexities and barriers of the health-care system. The CDRP, partnering with NIH/NCI’s Center to Reduce Cancer Health Disparities, was one of the first federal grants to formally add the requirement and funds to each grantee institution to establish the novel patient navigator position at each CDRP site.

In 2001 and 2002, 6 awards were made for a 5-year funding period, and an additional 5-year award was made in 2008 to 5 of the 6 original awardees (Table 1). Whether NCI cancer centers would respond to this model was uncertain; however, as shown in Table 1, most of the CDRP programs established relationships with both their potential mentors, indicating enthusiasm and commitment to this innovative program among the NCI cancer centers.

**Table 1.** Cancer Disparities Research Partnership Program programs

Primary program (awardee)	Primary partner (mentor)	Secondary partner (mentor)	Service area population	Target population
Rapid City Regional Hospital, Rapid City, SD	University of Wisconsin–Madison, Madison, WI	Mayo Clinic, Rochester, MN	300 000^a^	American Indian
Laredo Medical Center, Laredo, TX	University of Texas Health Science Center, San Antonio, TX	MD Anderson Cancer Center, Houston, TX	177 000^a^	Hispanic/Latino
Daniel Freeman Memorial Hospital, Inglewood, CA	University of Southern California, Los Angeles, CA	RAND Corporation, Santa Monica, CA	100 000^b^	African American Hispanic/Latino
New Hanover Regional Medical Center, Wilmington, NC	University of North Carolina at Chapel Hill, Chapel Hill, NC	—	616 000^b^	African American Urban/rural poor
Singing River Hospital, Pascagoula, MS	University of Alabama at Birmingham, Birmingham, AL	University of Mississippi Medical Center, Jackson, MS	200 000^b^	African American
UPMC, McKeesport Hospital, McKeesport, PA	Washington University, St Louis, MO	Roswell Park Cancer Center, Buffalo, NY	935 000^b^	African American Urban/rural poor/Amish

a Service area population as of fiscal year 2002 (October 1, 2001-September 30, 2002).

b Service area population as of fiscal year 2003 (October 1, 2002-September 30, 2003).

Funding for the CDRP ended in 2013. In 2014, Wong et al. (4) published an overview of and lessons learned from the CDRP program, including more details about the purpose, funding mechanism, implementation, and outcomes as of 2013. We endeavored to help the principal investigators seek funding, including participating in what is now the NCI Community Oncology Research Program (https://ncorp.cancer.gov/) as well as other NCI investigator grants, and provided information about non-NCI sources of funding.

This report provides an update—to the extent possible—primarily of the lessons learned and experiences of the awardees 10 years after the end of the CDRP grant program and over the 20 years since CDRP’s beginning. The successes and lessons learned from the awardees, the community-based institutions, and the academic mentoring institutions may be invaluable to those planning and implementing other health-care programs for communities experiencing health disparities.

## Methods

The intent for this 20-year report was to obtain an update from the principal investigators at the community-based and academic institutions on their experience in the CDRP and its impact on them and their communities. We attempted to gather this information through different avenues and from different perspectives. We hoped to obtain institutional data regarding the accrual of patients from the underserved communities enrolled in cancer clinical trials from the last few years, but this was not possible. A short survey was distributed to all 6 of the CDRP principal investigators, 5 of whom completed the survey and submitted responses. A similar questionnaire was sent to the awardee institution, but after numerous attempts to contact previous and current institutional administrators, we were successful in obtaining only 1 response, which was insufficient to include in this report. A 10-question survey using a 5-point Likert scale was distributed by email to all 6 of the CDRP principal investigators; space was provided for each question and at the end of the survey for additional comments. The survey questions are listed in Box 1; the response options were 1 = strongly disagree, 2 = disagree, 3 = neutral, 4 = agree, and 5 = strongly agree. The scores are an average of all responses received for that question.

Following the completion and analysis of the survey responses, the principal investigators were asked unstructured questions by email from the perspective of a decade after the end of the program to provide 1) what they had learned and 2) what they would have done differently. They were also asked to provide comments from those in the community who had participated in the CDRP.

We present a summary of the principal investigators’ responses to our survey and follow-up questions about their experiences with and lessons learned from the CDRP, using examples provided from the different principal investigators. This summary included narratives describing how the principal investigators’ participation in the CDRP affected their careers, institutions, clinical research, and communities. The importance of using narratives was exemplified in the CDRP relationship with the indigenous populations of the Lakota Sioux in the South Dakota CDRP Walking Forward program (5-8). Interestingly, a recent article from Australia pointed out the importance of “yarning and story-telling” between indigenous and nonindigenous populations (9). The narrative is an approach we in health disparities research now use in outreach to health-disparities populations in the United States and globally after having learned of their value in enhancing the knowledge obtained from our efforts to improve cancer care among underserved populations (8).

## Results

Five of the 6 principal investigators returned completed survey responses by email, an 83% response rate. Box 1 includes the summarized responses received, and the score for each question is an average of the respondents. To preserve anonymity because of the small number of responses, we reported neither the principal investigator names nor their relationship to the CDRP awardee or mentor institutions.

Box 1. Survey questions and responses from Cancer Disparities Research Partnership principal investigators^a^

**1. It was difficult to continue providing access to NCI cancer clinical trials for our patient population after the CDRP program ended because of funding issues (eg, no additional funding available, not enough funding).**

There was strong agreement (**4.6**) that following the end of CDRP funding, there was difficulty in continuing to fulfill the mission of the program to provide access to cancer clinical trials for their underserved populations because financial support is a necessity. For the Walking Forward program, which was successful during its first 10 years, the deficit of radiation oncology trials available through Radiation Therapy Oncology Group/NRG Oncology that underserved populations are eligible for remained a major impediment for enrollment in clinical trials (ie, clinical trial eligibility criteria because of preexisting co-morbidities present in these populations). Financial support and other required resources continue to be primary major barriers for the establishment of any CDRP program, especially when for-profit institutions have arisen to handle all patient care and exclude participation in clinical trials.

**2. Access to NCI cancer clinical trials for our underserved or health-disparate populations continued after the CDRP program ended.** *This question is specific to your community’s underserved or disparate populations and their access to NCI cancer clinical trials.*

There was neutral agreement (**3.0**) for why access to cancer clinical trials could not continue for their underserved populations after funding for the CDRP ended. Funding and resources continue to be impediments to continuation of cancer disparities accrual programs. Rapid City Regional Hospital has been more active in enrolling American Indians to cancer screening and social science studies than NRG Oncology treatment trials. UPMC participation in clinical trials in NCI’s National Clinical Trials Network continues to be limited because of patient ineligibility criteria, which remains to be addressed today for underserved populations. New Hanover Regional Medical Center’s small community-based health disparity research program has evolved through short-term participation as a Community Clinical Oncology Program awardee and NRG Oncology member and more recently with its affiliation with the Southeast Clinical Oncology Research Consortium within the NCI Community Oncology Research Program.

**3. The patient navigation component of your CDRP program has become an integral and important part of cancer care delivery at your hospital or health-care institution.**

There was strong agreement (**4.2**) for the various patient navigation programs established within each CDRP program. Rapid City Regional Hospital’s patient navigation continues today to be the cornerstone of the Walking Forward program because patient navigation is highly active on American Indian reservations. UPMC has continued with its patient navigation program to help identify vulnerable cancer patients for guidance on timely access to interventional therapies, while the most long-lasting and beneficial aspect of the CDRP at New Hanover Regional Medical Center remains patient navigation (ie, 1 person in radiation oncology, enhanced for multidisciplinary clinics).

**4. The CDRP program has changed your patient community’s expectations in terms of how and what patients now expect when they come for cancer care at your hospital or health-care institution.**

There was strong agreement (**4.4**) that the CDRP has changed each patient community’s expectation for cancer care at their institutions. Rapid City Regional Hospital became a known regional and national entity for community-based patient navigation once the underserved population became educated about this available program. For UPMC McKeesport, increased patient expectations have also been a result of the impact of the staff conveying this access availability to them. In the case of New Hanover Regional Medical Center, the CDRP raised awareness of service beyond clinical care by oncologists, including patient navigation and clinical trial opportunities. Receiving NIH awards was a important achievement for a small community hospital that led to numerous positive public relations and marketing campaigns.

**5. Your hospital or health-care institution is currently enrolling patients in NCI cooperative group trials (eg, NCI Community Oncology Research Program, NRG Oncology). This question is not just trials for health-disparate populations, as asked in question 3.**

There was neutral agreement (**3.6**) that former CDRP institutions are continuing to enroll their underserved patients onto NCI clinical trials. Lack of financial support drastically affects the staffing needed to continue enrollment, although limited enrollment to NRG Oncology and other NCI National Clinical Trials Network trials continues. New Hanover Regional Medical Center is currently enrolling patients in NCI cooperative group trials as a member of the Southeast Clinical Oncology Research Consortium NCI Community Oncology Research Program by working with the cancer registry for its patient population and target trials that appear feasible and most likely to recruit the largest number of eligible patients. The most challenging obstacle for enrollment remains the increasingly complex inclusion/exclusion criteria needed for patient eligibility.

**6. The TELESYNERGY system provided with the CDRP program was helpful in establishing the infrastructure needed to support cancer clinical trials at our hospital or health-care institution.**

Once again, there was neutral support (**3.0**) for the value of the TELESYNERGY system provided with the CDRP. It was predominantly underused but was critical for UPMC’s “mini-CDRP five sites system,” which allowed for the tumor boards, chart rounds, peer review, and treatment planning that continue today. The most successful use of TELESYNERGY was as a virtual meeting tool that enabled specialists (pathology, radiology, specialty surgery) to participate from partner institutions. Today, the system has been replaced by Zoom and WebEx.

**7. The CDRP program was beneficial in that it increased the number of physicians and staff at my hospital or health-care institution interested in specifically addressing cancer disparities and conducting health disparities research.**

There was strong agreement (**4.4**) that the CDRP had a lasting impact on the physicians and staff at the community-based institutions. The most successful—Rapid City Regional Hospital’s Walking Forward CDRP program—has blossomed into a 22-year program with 4 RO1 grants and multiple foundation grants. It is known regionally and nationally as an entity for community-based patient navigation for its American Indian population. The established trusting relationship with the tribal communities and Indian Health Service has been the foundation for its successful health disparity research program. UPMC has recruited staff (both clinicians and researchers) and engaged residents with specific interests in health disparities; it has gained the NCI’s support for continued engagement. At New Hanover Regional Medical Center, physicians are full-time clinicians and supportive of cancer and health disparities research, but their ability to be a research principal investigator and apply for NIH or other external awards is limited.

**8. The CDRP and patient navigation program focus on improving access to cancer clinical trials among underserved populations was used for other research and program areas at my hospital or health-care institution.**

There was neutral support (**3.0**) for CDRP/patient navigation programs being used at the community-based institutions once funding ended. Only the Rapid City Regional Hospital’s Walking Forward program succeeded in accomplishing this because of the commitment/perseverance of Principal Investigator Dr Dan Petereit and his health disparity colleagues and collaborators. His continued publications on all aspects of the Walking Forward program has earned regional and national status as a model for addressing cancer health disparity for their American Indian population. UPMC’s hybrid tumor registrar/navigator has also continued to be used for the social work intervention its underserved populations need.

**9. The CDRP program altered how the grantee institution approached health disparities in our community.**

There was strong agreement (**4.2**) on how their CDRP program affected the institution’s approach to health disparity for their community. It is reassuring to see that Monument Health in Rapid City, SD, has embraced Rapid City Regional Hospital’s cancer disparity model, which Walking Forward developed. Although now administratively part of the Avera Health System in Sioux Falls, SD, Monument Health and Avera Health in Sioux Falls are close collaborators and provide many patients living on the reservations in western South Dakota and Rapid City with an opportunity to be enrolled in clinical trials. UPMC has continued its outreach and health screening efforts, expanding it to include the Amish community (not originally in the grant). The continued use of the TELESYNERGY system for tumor boards and videoconferencing validate the system’s importance, especially during the pandemic. New Hanover Regional Medical Center’s community-based disparity program has confirmed the importance of increased awareness of and need for community outreach.

**10. The CDRP program altered the career path of the principal or senior investigators who participated in this program.**

There was neutral agreement (**3.2**) about the CDRP program altering the principal investigator’s career path and goals. For Dr Daniel Petereit, the Walking Forward program resulted in a major career path alteration, both personally and professionally. This program allowed an academic program at the University of Wisconsin to implement NIH-funded clinical trials in Rapid City, SD, for the first time in 22 years. Dr Dwight Heron continues to be immersed in health disparities research both domestically and internationally and was the institutional principal investigator of the University of North Carolina’s ACCURE grant, funded by the NCI. Dr Michael Steinberg has continued his academic pursuits and became chair of Radiation Oncology at the University of California Los Angeles, where he continues to address issues of health equity, including leading a University of California Los Angeles effort to return to the community that the Los Angeles CDRP program served by establishing a community-based academic radiation oncology center for training and research serving the community. For Dr Patrick Maguire, being a CDRP principal investigator probably played a part in his placement on the New Hanover Community Endowment Board, with a $1.25 billion fund dedicated to increasing the wellness, education, safety, and economic prosperity of the people in New Hanover County, NC. Although the short-term CDRP program helped his co-investigators be recognized, Dr Yadvindera Bains (as a principal investigator heavily interested in disparities and clinical trial access for patient from minority and underserved communities) remained in private practice, away from academic politics.

^a^CDRP = Cancer Disparities Research Partnership Program; NCI = National Cancer Institute.

Of the averaged responses, there was strong agreement (4.6) that it was difficult to continue providing access to NCI cancer clinical trials after the CDRP ended (question 1) and agreement (4.4) that the CDRP changed the patient community’s expectations for cancer care (question 4) and was beneficial in increasing the number of staff and physicians interested in and conducting health or cancer disparity research (question 7). Also, there was agreement (3.6-4.2) that the patient navigation program was integral to cancer care delivery (question 3), that their institution is currently enrolling patients in NCI cooperative group trials (question 5), and that the CDRP altered how the grantee institution approached health disparities in the community (question 9). There was neutral agreement (3.0-3.2), however, for questions about continued access to NCI cancer clinical trials for their health-disparities populations (question 2), that TELESYNERGY was helpful in establishing infrastructure to support cancer clinical trials (question 6), that the patient navigation programs were used for other program areas or research (question 8), and that the CDRP altered the principal investigators’ career path (question 10).

As part of the survey’s open-ended comment field, 5 principal investigators shared their thoughts on the CDRP and the future, summarized below. Interestingly, sustainability of funding, longer commitment by funders and health-care administrators, and demonstrating the value of clinical research in community hospitals and settings not normally involved in research is still needed, as evidenced by these 2 responses:

- ***How would I structure my program going forward?*** “We should ensure long-term commitment (beyond the duration of federal grant funding) to clinical cancer research from community hospital administrations via a matching funds pledge or similar mechanism.”
- ***How would I improve sustainability?*** “The need for continued funding needs to be stressed. To elaborate more: most decision-makers in community hospitals have minimal insight into the benefits of clinical research. They view it as bleeding red financially, in light of high personnel costs vs moderate direct reimbursement for patient enrollment onto NCI-sponsored trials. It is the rare hospital administrator who grasps the next-level competitive advantages that the addition of NIH clinical trial offerings may bring. One answer to this problem may be enhanced use of telemedicine for centralized enrollment of patients, particularly those in rural and/or underserved populations, onto clinical trials.”

After considering the survey results, we asked the principal investigators supplementary questions. The following are the summarized responses we received:

- ***What are the lessons learned at the end of the CDRP program and from another decade of observation?*** We learned from the 5 responding principal investigators that 1) 2 had sustained funding—1 through NCI Community Oncology Research Program and another through sustained grant support (Walking Forward). For the other programs, when the funding ran out, the programs disappeared. 2) The CDRP changed the expectations of the patients’ community and increased interest among physicians, staff, and health-care institutions in addressing health disparities. 3) The patient navigation component became an integral part of some institutional cancer programs. 4) The CDRP altered how the awardee institutions approached health disparities. Thus, although anecdotally the CDRP appeared to have an impact, in addition to the establishment of patient navigator programs, increasing community and physician/staff awareness of the benefits of patient navigation was the most obvious success resulting from the CDRP.
- ***If we designed a CDRP grant today, what if anything would we do differently than we did 20 years ago?*** We learned that first we would incorporate long-term metrics for quantifying the CDRP’s impact on patient accrual into cancer clinical trials and of which type. Second, we would consider other potential outcomes, supporting “a value-added proposition” for the CDRP. Incorporating these 2 points into a future CDRP may help build sustainability into the program. We believe that if the CDRP had been sustained financially, it would have resulted in a greater and broader impact on increasing recruitment into clinical trials among these underserved populations. Further, although the CDRP emphasized participation in cooperative group trials (and had some success doing this), the under-representation of health-disparities populations is most prevalent and widespread in pharmaceutical industry–sponsored drug registration studies; we believe that greater effort to address this problem is necessary (10,11).

From the 1 sustained program, Walking Forward, we were able to obtain comments from members of the community who were patients, family members, and patient navigators (Box 2).

Box 2. Walking Forward patient, patient navigator, and staff testimonials, 2023

“The services provided by the program were very helpful. They provided P— the ability to get the treatments that she needed. We were and I am so grateful for the care and compassion shown by the people in the Walking Forward program. The program is an asset to the community and provides peace of mind. Thank you for the opportunity to verbalize my feelings.”

      —Walking Forward patient’s family member

“As a patient navigator I witnessed some remarkable outcomes. I witnessed patients having someone take away the worries of transportation to treatment, housing during treatment, food costs, and treatment costs. [I witnessed] patients and families having peace of mind knowing they had an advocate for them throughout their treatment regimen. [I witnessed] patients and families having someone they could talk to, to address their concerns and be a friend in a strange and scary place in their lives and be a rock they could lean on when needed throughout treatment. This is just a glimpse of what I witnessed as a patient navigator.”

      —Former Walking Forward patient navigator

“Growing up in a rural poverty-stricken community being a patient navigator with Walking Forward was and is still one of the most rewarding jobs I have had. I will forever cherish having the opportunity to provide guidance and hope for my relatives at a time when they were most vulnerable. My father was one of them, being diagnosed with end stage lung cancer, our family was relieved of the many stressors that come with navigating and enduring that diagnosis. We are forever grateful.”

      —Former Walking Forward Rosebud Reservation Community Navigator and patient’s family member

“The Walking Forward helped save my life. This program and the people involved in it genuinely made me feel hopeful in a hopeless situation. They reduced any barriers that impeded my treatments and helped me stay on track for my appointments while offering compassion and emotional support.”

      —Former Walking Forward patient

“Walking Forward patient navigator (WF PN) Program has helped bridge the gap for many cancer patients who get referred to other facilities for their care. With the Reservation having one of the highest unemployment rates in the country and other unmet needs, WF PN Program was able to assist many cancer patients with financial assistance for food, gas and a motel stay, along with navigation for appointments.

“As a Navigator I have been told numerous times from patients that if it were not for WF they may have not been able to attend all appointments, treatment options, and follow up visits in a regularly scheduled timeframe. Many have expressed that they wished this program existed when their parents/grandparents were diagnosed with cancer, maybe they would have lived longer. One patient said not only did he fear a cancer diagnosis but also the fear of how will I be able to afford the cost of traveling to a city 3-4 hours away for 8 weeks.

“Over the course of 11 years WF is recognized on the Reservation as a major contributor to helping patients get the care that they need in order to complete their treatment plan. I think the highest compliment WF received was that patients felt like they could finally trust in their treatment plan knowing that WF will provide navigation. One patient asked if he could change facilities so that he could be part of the WF Program. The Native Community holds WF in high regard. WF has proved to be consistent and visible in the community. The Native Community is grateful and blessed for WF presence.”

      —Walking Forward Rosebud Reservation community navigator

“At a recent American Indian Cancer Survivor Conference in Rapid City, SD two former cancer patients who were part of the Walking Forward patient navigation project during their treatment in Rapid City, each shared how the program impacted their cancer treatment experience while sharing their survivor stories. They both stated that the WF program helped alleviate their worries and financial concerns, especially regarding the logistics of getting to their treatment. Having this type of support available made a difference for them in being able to complete treatment.”

      —Walking Forward Rapid City community navigator

## Discussion

The CDRP was a transformative and novel model of grant funding, with evidence of persistent impact more than 12 years since the end of funding. Patient navigation, community outreach, a culture of clinical trials, and associated infrastructure are lasting elements dependent on institutional commitment. One program has sustained continuous funding from both NIH and nongovernment sources, and another joined the NCI Community Oncology Research Program. That said, health disparities remain, and they became even more visible during the COVID-19 pandemic, further illustrating the continued need for financial investments in this area.

The primary purpose of the CDRP was understanding and increasing the limited enrollment of populations facing health disparities onto NCI clinical trials. The CDRP also emphasized radiation oncology for the technical and computer expertise and image-heavy practice to pioneer TELESYNERGY. Radiation oncology clinical trials were emphasized, although broader participation in cancer prevention and treatment trials was also encouraged. Radiation oncology was considered extremely important in populations experiencing health disparities because they tended to present with more advanced-stage cancers, which often required radiation as a component of their treatment. Because treatment courses are often multiple weeks in duration and access to radiation therapy is essential for effective cancer care, the issue of how to enable completion of a course of treatment was encountered, as anticipated.

The current emphasis on health-care disparities by addressing equity, diversity, and inclusion is likely to be beneficial, but it must be recognized that short-term to medium-term funding must be made available in concert with a true long-term funding strategy for benefits to be sustained. “Moonshot” strategies garner interest and have impact, but what happens at “the end” of a bolus of funding when the funder absorbs these new projects into its overall research system is a challenge. Based on broad interest and the experiences of several co-authors in the global health and global cancer care communities, we see that cancer disparities in economically advantaged countries are similar in many ways to that in low- and middle-income countries. “Local-global” aptly demonstrates the similar problems and challenges that populations experiencing disparities face in access to and use of cancer care and clinical trials as well as shared solutions (12). Such recognition may raise the interest in and support for career paths in both global health and equity, diversity, and inclusion (13-15).

Accomplishments from the 2014 analysis of the CDRP relative to the 20-year follow-up are presented next, with further details available in Wong et al. (4). For NCI cooperative group cancer clinical trials, few of the clinical trials had eligibility criteria suitable for participation by patients with the advanced-stage disease that CDRP awardee and mentor institutions frequently encountered. To rectify this, CDRP principal investigator–initiated and mentor-initiated clinical trials were pioneered and underwent ad hoc peer review. These trials were successful in accrual but were discontinued because they required a formal, ad hoc review by NCI and CDRP-only approval was not deemed sufficient. Efforts to activate trials for more advanced-stage disease were generally not successful in the cooperative group structure because they did not receive sufficiently high priority. A key feature of the CDRP was piloting the patient navigation program. Energized and led by Dr Freeman (3), patient navigation enhanced patient participation and reduced the number of “missed treatments,” which was important for radiation therapy care and institutional costs. This approach was successful and remains in place in several facilities, often at their own expense. Further, regarding the general experience of the CDRP, the communities responded positively to committed principal investigators, but there was mixed long-term impact, with some programs remaining strong based on future grants and partnering with interested health-care systems. Patient navigators and survivorship programs became part of the community following the grant program. The critical importance of community engagement was demonstrated by the Daniel Freeman Memorial Hospital CDRP team in California (16). This awardee’s program was a transformational change, yet it ended with closure of the hospital.

Critically, it can take years before the necessary trusted relationships are established between investigators and the community. Funders, grants, and contracts must take this time requirement into account when assessing progress and continued support. Similarly, the commitment to research programs varied because of the reality of underserved regional hospitals. Quality research is essential to be competitive for NCI funding, but it took years for some awardee community institutions to get up to speed with the grant and research systems. Other mechanisms of support beyond the time-limited grant process would be critical. Starting and discontinuing programs are disruptive to all involved and can lead to a lack of the trust essential in reaching these underserved communities. In addition, other metrics for assessing “career productivity” for the investigators would be valuable because the standard metrics of patient accrual, number of publications, and “impact” are much more difficult than what is possible in an academic center or affiliated practice. Academic mentors embraced the opportunity. Finally, the TELESYNERGY system was useful for mentor-mentee discussions and for the CDRP investigators and NCI staff to hold meetings. When 1 of 2 potential mentees was selected, the unselected mentee also participated enthusiastically, which speaks to the CDRP fulfilling an important gap for several major radiation oncology programs.

Regarding a primary goal of access to cooperative group cancer clinical trials, little has changed. Few of these types of trials have eligibility criteria suitable for the advanced stages of disease underserved populations and institutions frequently encounter. The lack of community participation appeared to be an issue of availability of clinical trials rather than a lack of interest or willingness.

Community is key. The community will respond to committed community principal investigators and support research initiatives. In addition, survivorship and advocacy programs are more commonplace in many health-care settings and institutions. Communities in the CDRP areas have higher expectations for cancer care because of the CDRP. Participation in research has been variable and community specific.

It takes time. It can take several (perhaps many) years before the necessary trusted relationships are established with and between investigators, the awardee institution, and the community. Funders (particularly grants and contracts) need to understand and consider that the relationship-building “startup time” is extremely important and allocate sufficient time for this step when developing timelines, benchmarks, and outcomes. For example, the NIH grant system (and others) may need to adapt to this reality and allow the lead time necessary not only to launch but to sustain research studies. Notably, understanding exactly how trusted relationships are built and sustained in research is, in itself, research. Providing “gap funds” to allow disparity grantee institutions reasonable opportunity to obtain funding to sustain the program is critical, particularly given the challenging percentiles for successful NIH funding. Additional funding streams beyond the NIH are necessary for this type of research, and community involvement is imperative.

Telemedicine became commonplace during the COVID-19 pandemic, with the virtual nature of health-care visits shown to be necessary because of the pandemic. How this will be continued remains to be seen, but likely this is now an embedded feature in health care for remote and other underserved communities.

Finally, the impact on participants in the CDRP was positive. Participation by community members in on-the-ground programs to address disparities in cancer clinical trials was generally positive. Unfortunately, the ability to continue this type of research and work as part of a career path is limited by the current funding environment and lack of opportunities. It appears to be increasingly difficult for faculty at academic institutions with or without health-care systems to participate in research and work on addressing disparities in cancer care without receiving external funding. One idea is that other metrics for assessing “career productivity” might enable health disparities research and community efforts to become an integral part of an academic researcher’s career. This change would, however, require academic pathways to promotion and institutional commitment to activities that are not necessarily revenue generating.

Looking to the future and considering the lessons learned from the CDRP’s 20-year experience, we considered and addressed 2 questions.

### Can CDRP efforts to increase participation in clinical trials among populations experiencing health disparities be generalized?

With the premise that randomized controlled trials are critical to delivering sustained improvements in cancer care, Dodkins et al. (17), Wilson and Rodin (18), and Dee et al. (19) identified a need for more randomized trials in low-income and middle-income countries and for trials to address the types and stage of cancer most experienced in these countries. This global need is in concert with a finding from the CDRP for access to clinical trials that includes the development of infrastructure expertise and financial support. This finding represents a generalized conceptual approach, linking equity in cancer care within resource-limited settings in high-income countries (eg, CDRP sites) and low-income and middle-income countries, with the similarity referred to as “local-global” (12).

We also now have a better understanding of the many and varied challenges these underserved populations experience in meeting their comprehensive health-care needs. Many of these challenges fall outside the scope of the CDRP, however. For example, reducing disparities in these underserved communities should not be limited to allowing members access to clinical trials. Members coming from these underserved communities should also be involved in prioritizing the research needs of their community and designing the trials and health care that meet the needs of this population. Unfortunately, huge workforce realities limit the feasibility of members from these communities meeting these needs (20-22). For example, Janopual et al. (22) reported that in 2019, there were 853 senior radiation oncology faculty, with 508 (60%) reported as White, 279 (33%) claiming Asian ancestry, and only 13 individuals (1.5%) categorized as Black. With such a small number of Black investigators spread throughout the country, it would be extremely challenging for them to have a meaningful impact on addressing the health disparities that nearly 42 million Black Americans experience. Addressing the composition of the radiation oncology workforce would be an important, complementary component of any future programs attempting to increase participation among underserved populations into clinical trials (21). Such a program might incorporate this facet into its own design or closely align with a separate program focused on building, strengthening, and maintaining the necessary workforce and appropriate racial and ethnic representation.

### How can a better future be assured?

The keys to addressing the long-standing disparities underserved communities experience must include community empowerment and sustainable programs and funding. The former challenge means that we must facilitate and encourage the engagement, recruitment, and mentorship of young people to pursue careers that will enable them to drive the directions, themes, and strategies required to ensure that their own communities are protected against the social injustices that led to their health disparities (20,22). The latter challenge requires that funding agencies (such as the NCI) value and pursue longer financial commitments to make meaningful and sustained changes for these underserved populations. Some of these issues are currently addressed in part by Center to Reduce Cancer Health Disparities’ Continuing Umbrella of Research Experiences program (23), its Early Investigator Advancement Program (24), and the Partnerships to Advance Cancer Health Equity program (25). For both challenges, the criteria for success must be based on the reality of the prolonged time needed for the programs to succeed and the negative effect on all parties (researchers, patients, communities) of starting, then abruptly ending. It is proposed that there be appropriate criteria for success, for the investigators to sustain a career path addressing disparities in health care, and for institutions to receive recognition for their efforts.

## Conclusions

The CDRP principal investigators and program directors are grateful to the visionary leaders at the NCI, who were willing to support an approach that was novel at the time. We appreciate most of all the wisdom and efforts of the institutions, patients, families, and communities who helped pioneer this effort.

There are limitations to this 20-year retrospective. First, we were unable to obtain institutional data regarding the accrual of patients from the underserved communities enrolled in cancer clinical trials from the last few years because of patient privacy and confidentiality concerns. Second, we made numerous attempts to contact previous and current institutional administrators to send our survey but were successful in obtaining only one response, which we were unable to use. Finally, we inquired with NCI officials regarding clinical trials participation data from grantee institutions; these data were confidential and so not available.

The CDRP was designed more than 2 decades ago to improve accrual for health-disparate populations onto NCI clinical trials. The CDRP successfully piloted a unique funding model—one that reversed the typical flow of funds to the awardee institution based in the community and directly served health-disparate populations from the NCI cancer centers. NCI cancer centers were eager to be partners with the CDRP awardees and their network.

We conclude that investment in research infrastructure, research staff, and logistical support is critical to success, and these transformative grants take more than 5 years to build institutional research milieu with a sustained research structure. Most importantly, it takes a long time to establish trust among researchers and the populations in communities they serve.

The CDRP demonstrated that the communities experiencing the greatest health disparities play an important role by sharing their experiences, ideas, wisdom, and willingness to engage with those pioneering new approaches to address chronic problems. The wise amalgamation of top-down expertise, bottom-up real-life experience, community participation, and listening to one another is a way forward, all while recognizing that progress will be slow compared with basic research and require a longer time before showing impact and reductions in disparities.

## Data Availability

The data underlying this article cannot be shared because of the privacy of individuals who participated by providing their personal experiences and opinions in the study. We provided summary-level data in the manuscript of the participants’ responses.
